# Biotherapeutic protein formulation variables influence protein integrity and can promote post-translational modifications as shown using chicken egg white lysozyme as a model system

**DOI:** 10.1007/s10529-015-2014-y

**Published:** 2015-12-23

**Authors:** Evdoxia Gourbatsi, Jane Povey, Shahid Uddin, C. Mark Smales

**Affiliations:** School of Biosciences and Centre for Molecular Processing, University of Kent, Canterbury, CT2 7NJ UK; Formulation Sciences, AKB Building, MedImmune, Granta Park, Cambridge, CB21 6GH UK

**Keywords:** Protein formulation, Mass spectrometry, Post-translational modification, Aggregation, Peptide model

## Abstract

**Objectives:**

The effect of different formulations variables on protein integrity were investigated using lysozyme as a model protein for the development of biotherapeutic protein formulations for use in the clinic.

**Results:**

Buffer composition/concentration was the key variable of formulation reagents investigated in determining lysozyme stability and authenticity independent of protein concentration whilst the storage temperature and time, not surprisingly, were also key variables. Tryptic peptide mapping of the protein showed that the modifications occurred when formulated under specific conditions but not others. A model peptide system was developed that reflected the same behavior under formulation conditions as intact lysozyme.

**Conclusions:**

Peptide models may mirror the stability of proteins, or regions of proteins, in the same formulations and be used to help develop a rapid screen of formulations for stabilisation of biotherapeutic proteins.

**Electronic supplementary material:**

The online version of this article (doi:10.1007/s10529-015-2014-y) contains supplementary material, which is available to authorized users.

## Introduction

The number of biopharmaceutical protein-based drugs on the market and in development continues to increase with biopharmaceuticals making up a significant portion of new drugs in the development pipeline. Protein based drugs are susceptible to degradation and aggregation (Roberts 2014) that compromised integrity and as such must be carefully formulated after their expression and purification at the appropriate pH, appropriate concentration, in the appropriate buffers and with appropriate stabilising excipients to prevent unwanted degradation, modification and aggregation events (Mitragotri et al. 2014). The development of the “best” formulation for a given biotherapeutic protein to preserve its integrity largely occurs using a knowledge based, design of experiments trial and error approach using biophysical methods to determine, amongst other parameters, how formulation variables influence aggregation and protein stability (Chaudhuri et al. 2014).

In order for biopharmaceutical drugs to be successful in clinical applications, appropriate formulation(s) for preservation, stability and delivery need to be determined. This is not an easy task as each protein biopharmaceutical is unique and small differences in the amino acid residues result in the need of a specific formulation to deliver maximal stability and activity for each protein. Preservation is usually investigated using elevated temperature and varying pH in order to ‘force’ stability issues. Lysozyme, whilst not a therapeutic protein, is a well-characterised protein molecular making it a good protein to investigate the influence of formulation variables on protein stability and has previously been used for such purposes (Povey et al. 2009; Smales et al. 2000; 2001). The effect of different formulations variables on protein integrity were therefore investigated using lysozyme as a model protein for the development of biotherapeutic protein formulations for use in the clinic.

## Materials and methods

### Reagents

All reagents, including egg white lysozyme (L6876-5G, lyophilized powder) were purchased from Sigma-Aldrich and were of analytical grade or better.

### Plackett–Burman design of experiments

The effect of formulation variables (pH, buffer composition (mM), time (h), temperature (°C), glycine and NaCl concentration) on lysozyme solubility/aggregation and activity studies were investigated using a Plackett–Burman Experimental Design (based on Zhao et al. 2005) (Supplementary Tables 1 and 2). Triplicate samples were investigated for each treatment and the mean calculated for data analysis. A two tailed *t* test was used to compare the two sample means i.e. the low and high values of each variable.

### Preparation of protein samples in appropriate formulations

The lysozyme samples (low 0.07 mM and high 0.81 mM) were prepared and then dialyzed against two changes of the appropriate formulation; one for 2 h and one overnight. After incubation in the appropriate conditions, samples were centrifuged at ~200×*g* for 4 min in a Eppendorf centrifuge. The pellets were carefully separated from the supernatants and resolubilised in 100 µl 8 M urea/0.25 M Tris/HCl buffer (pH 8.75)/1 mM EDTA. The concentration of the initial supernatant and solubilised pellet was determined by measuring the A_280_.

### Lysozyme activity assays

The activity of lysozyme was measured using *Micrococcus lysodeikticus* as a substrate using the method previously described (Povey et al. 2007).

### Tryptic peptide mapping

Lysozyme samples were subjected to tryptic peptide mapping using the method previously described (Smales et al. 2000).

### Data analysis

All data was analyzed using the Sequential Design of Expert tool (EasyStats, DX7, Version 7.1.6) to investigate and correlate the effect of individual variables and predict the best formulation conditions for long term storage at 4 and 25 °C.

### Liquid chromatography-electrospray ionization mass spectrometry analysis of intact lysozyme and tryptic peptides following incubation in different formulation conditions

Mass spectrometry analysis of the intact lysozyme samples after incubation in the different formulations and the tryptic digest samples were undertaken as previously described (Smales et al. 2000). To identify potential amino acid modifications a peptide that was close to the native chicken lysozyme peptide T12 + 13 but contained a modification in the third residue (I at position 3 changed to P) was synthesised to give a final sequence: SDPTASVNCAKKIVSDGNGM (MW: 1992.92 Da). 0.81 mM samples were prepared in PBS pH 7.3 (used as the standard/control sample) and formulations 1, 4 and 12 (Supplementary Tables 1 and 2). After incubation in the appropriate conditions, the pellets were carefully separated from the supernatants and the pellets resolubilized in 100 µl 8 M urea. Samples were diluted to 2 μg/μl using H_2_O with 0.05 % TFA and mass spectrometry analysis undertaken using a microTOF-Q II™ ESI-qTOF mass spectrometer (Bruker Daltonic GmbH) coupled to an HPLC. The analysis and identification of possible modifications to the peptide was undertaken using the PAWS EXE protein analysis program (ProteoMetrics) and Delta Mass database of protein post translational modifications (http://www.abrf.org/index.cfm/dm.home).

## Results and discussion

### Quantitative determination of lysozyme solubility and aggregation in different formulations

All the formulation variables, concentrations and levels used in this study were based upon those reported in previous studies (Trikha et al. 2002; Walsh 2006; Wang et al. 2007). The concentration of protein before and after incubation in solution was determined by measurement of the A_280_ (Supplementary Table 3). The A_280_ values were measured immediately after formulation and again after the relevant incubation time. A decrease in the A_280_ value and soluble protein is indicative of aggregation/precipitation of the protein and loss of protein in solution. Based on previous studies using a Plackett- Burman approach (Domart-Coulon et al. 1994; Zhao et al. 2005) if the statistical significance of a variable was greater than 80 % it was considered a significant factor.

Significant changes in A_280_ measurements were calculated as absolute amounts (mg/ml using extinction coefficients) and then as a % of the original compared to a PBS control (Supplementary Table 4). In all high concentration formulations, less protein was soluble than in PBS alone and in the case of formulations 1 and 12 there was a > 40 % loss in soluble protein relative to the PBS standard formulation. This was less prevalent in low concentration formulations although formulations 2 and 3 had a > 30 % loss in soluble protein compared to the control (Supplementary Table 4). The majority of protein aggregation occurred upon formulation and not during the following incubation period (Supplementary Table 4). From the A_280_ analysis and two tailed t-test statistical testing (Supplementary Table 4), the buffer composition was found to be statistically the most significant variable influencing soluble protein concentration followed by the protein concentration upon initial formulation. Following incubation under the different conditions, the most significant factor in terms of influencing soluble protein levels was the time of incubation (time of storage after formulation). Buffer composition was therefore confirmed as being a key determinant of aggregation, the formation of which can influence product performance and must be controlled during formation of biotherapeutics (Roberts 2014).

### The effect of formulation variables on lysozyme enzymatic activity

**Fig. 1:**
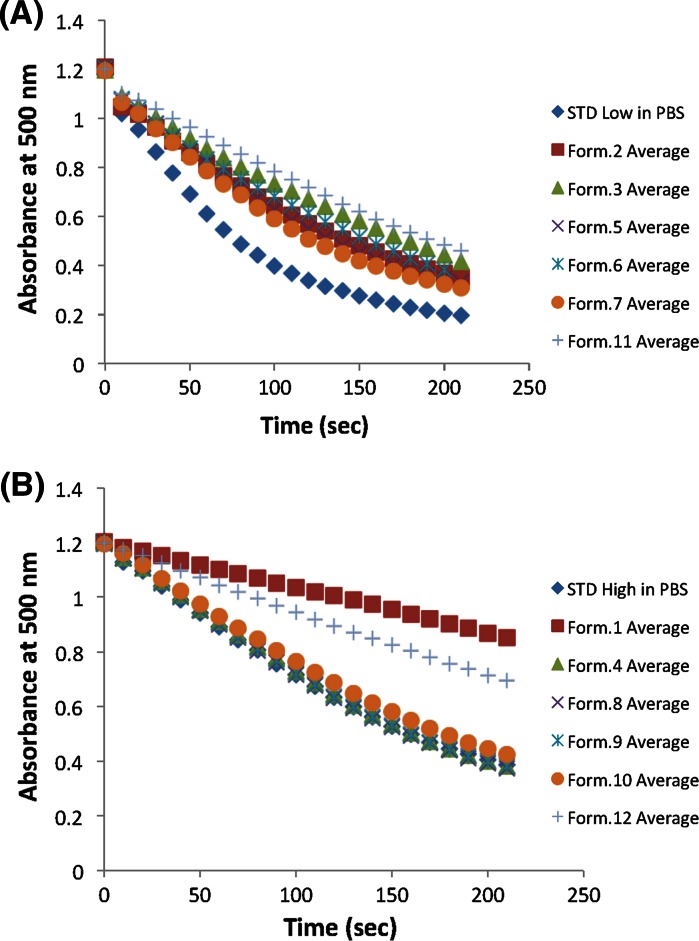
Absorbance clearing curves of lysozyme samples of different formulations using the bacterial substrate *Micrococcus Lysodeikticus* (n = 3). **a** Low lysozyme concentration samples, **b** High lysozyme concentration samples

Lysozyme initial rate activity was determined by measuring the OD_500_ of a suspension of the substrate *Micrococcus lysodeikticus* in the presence of lysozyme in each formulation (Fig. 1). (It is noted that the amount of each sample added to the assays was not sufficient to change the ionic strength or pH of the solution, which could influence the observed lysozyme activity.) Most of the formulations had no effect on lysozyme initial clearing rate activity although the rates were reduced in formulations 1 and 12 compared to the standard lysozyme sample in PBS (Fig. 1, Supplementary Tables 5 and 6). Statistical analysis showed that buffer composition was the variable with the greatest influence on the initial rates observed (Supplementary Table 6).

The equivalent of V_max_ and K_m_ of lysozyme samples in different high concentration formulations (formulations 1, 4, 8, 9, 10 and 12) was also determined and the resulting data in Supplementary Table 7 shows that the protein activity is dramatically affected by all formulation variables when compared to the control in PBS. Three of the formulation conditions (4, 8, 9) showed a small change (decrease) in initial rate compared to the control but the maximum clearing rate and concentration of substrate at ½ maximum clearing rate were much reduced compared to the control (Supplementary Table 7). The formulations that showed the most aggregation by A_280_ measurements (formulations 1, 10, and 12) showed a large drop in initial rate, and the biggest change (decrease) in the maximum clearing rate and concentration of substrate at ½ maximum rate confirming that these formulations were detrimental to enzymatic activity (Supplementary Table 7).

### Direct ESI–MS analysis of intact lysozyme for protein modifications under different formulation conditions

**Fig. 2:**
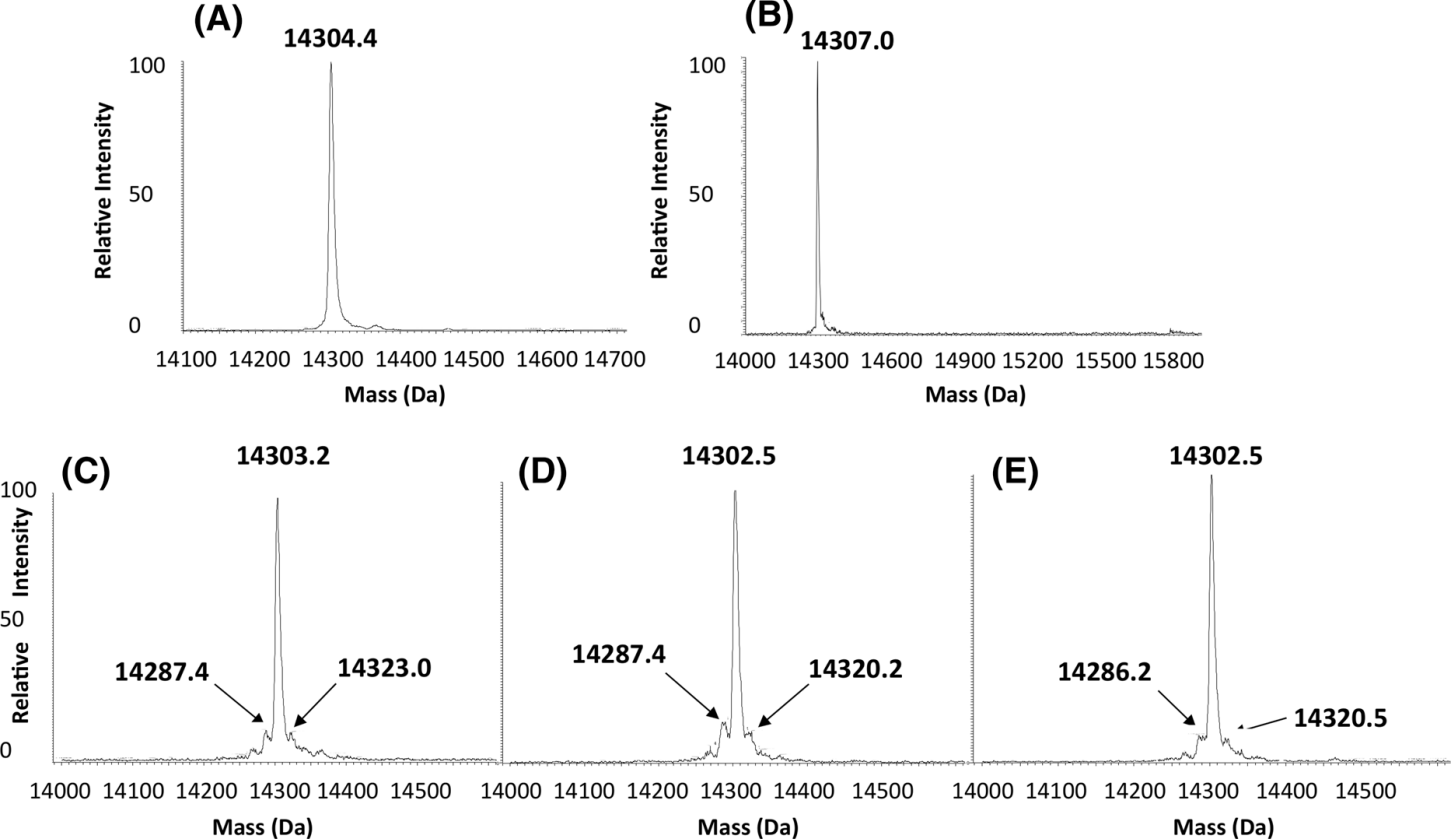
ESI-MS analysis of intact lysozyme under different formulation conditions. **a** Standard sample in PBS, **b** Sample formulation 2 supernatant, **c** Sample formulation 3 supernatant, **d** Sample formulation 6 supernatant and **e** Sample formulation 11 supernatant

**Fig. 3:**
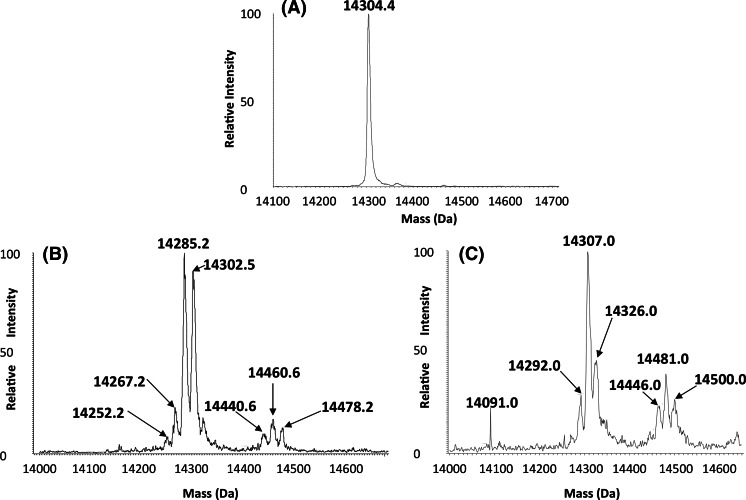
ESI–MS analysis of intact lysozyme in formulation 12. **a** Standard lysozyme sample in PBS, **b** representative lysozyme formulation 12 supernatant sample after incubation in formulation 12, and **c** representative lysozyme formulation 12 pellet after incubation and resolubilization in 8 M urea solution

As shown Fig. 2, a peak that corresponds to that expected for lysozyme (14,307 Da) was dominant in the control and formulation 2 samples indicating no observable and stable modifications occur in this formulation. In contrast, in the low protein concentration formulations 3, 6 and 11 (Fig. 2c–e) additional peaks were observed. A peak before the main lysozyme peak of mass 14,287, 14,287 and 14,286.2 for formulations 3, 6 and 11 respectively was observed, this loss being prevented in formulation 2. On the other hand, an additional peak after the main lysozyme peak of a mass 14,323.0, 14,320.2 and 14,320.5 in formulations 3, 6 and 11 respectively, corresponds to a gain in mass of 17–20 Da, which approximately equates to the gain of a water molecule. Mass spectrometry analysis of lysozyme samples in formulation 12 showed many changes in mass to the protein after incubation in this formulation (Fig. 3). The major peak in the supernatant had a mass of 14,285.2 Da, a loss of 17 compared to the standard sample, which could to be due to the formation of pyroglutamic acid formed from Gln or succinimide formation from asparagine (loss of 17 Da).

### Tryptic peptide mapping and reverse-phase HPLC–ms analysis of lysozyme formulations

**Fig. 4:**
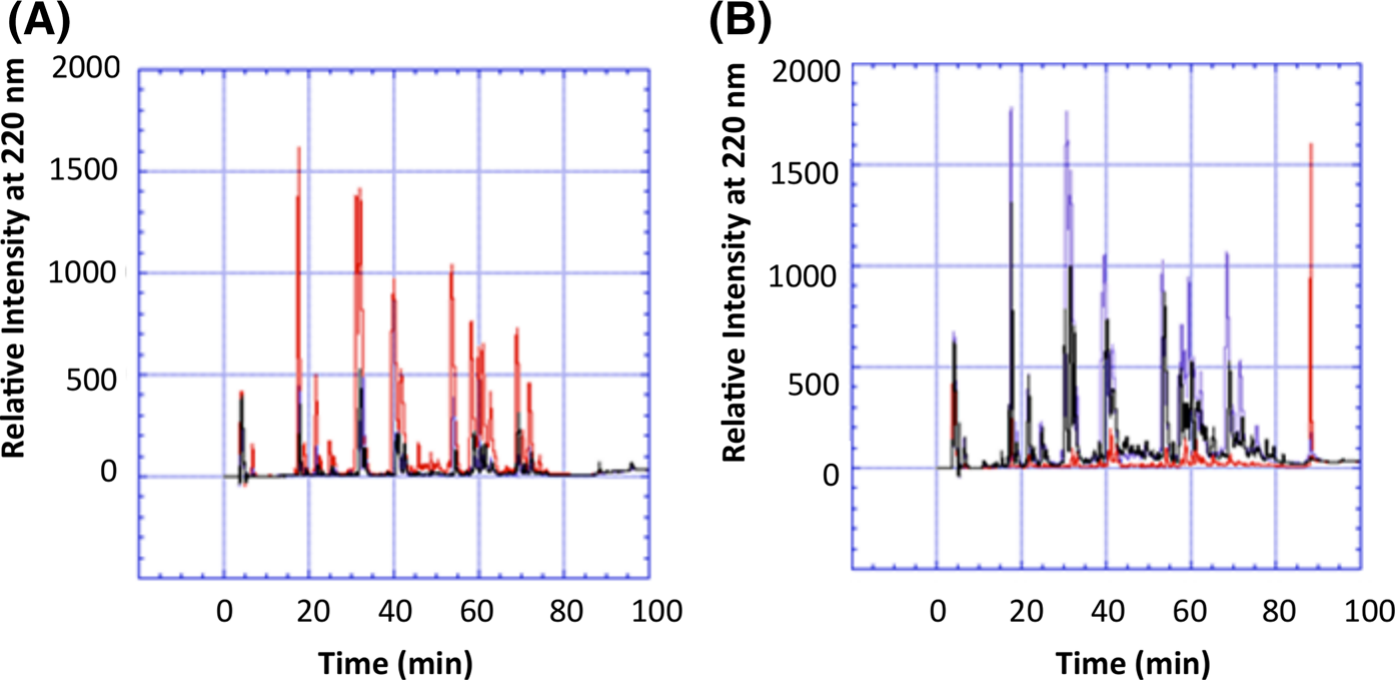
Separation of lysozyme samples in different formulations by reverse-phase HPLC on a C18 column after trypic digestion. **a** Lysozyme supernatant samples after incubation in formulations 1 (*red*), 4 (*blue*) and 12 (*black*), **b** Lysozyme pellets samples after incubation in formulation 1 (*red*) and 12 (*blue*) and lysozyme pellet sample in formulation 12 (*black*) before incubation

**Fig. 5:**
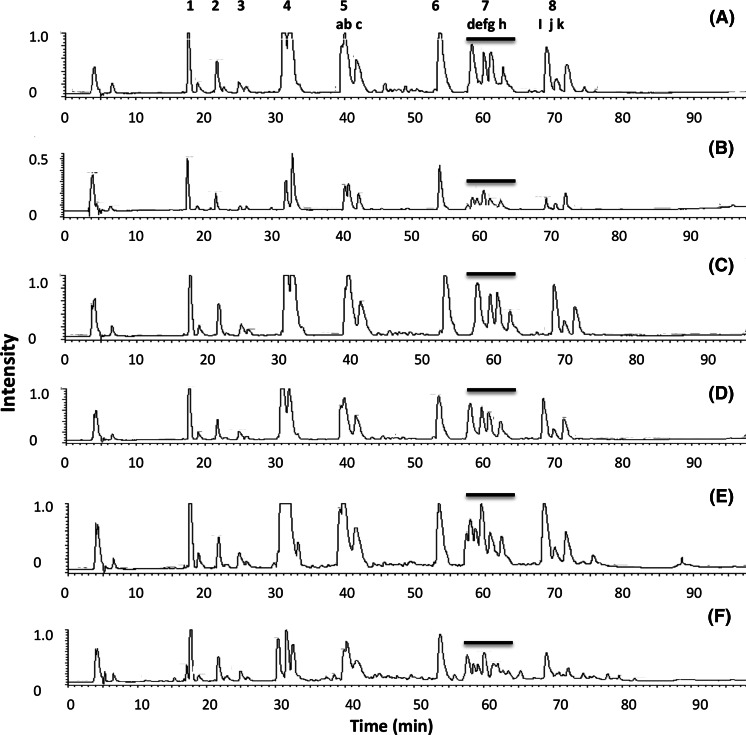
ESI–MS analysis of lysozyme in the various formulations after tryptic digestion. The *black bar* shows the area of peptide T12 + 13 (uncleaved peptide), which is known to exist in several forms. This area in particular shows changes with formulation suggesting it is susceptible to modification in agreement with previously published studies. **a** Formulation 1, **b** formulation 4, **c** formulation 8, **d** formulation 12 supernatant, **e** formulation 12 pellet after incubation and **f** formulation 12 pellet before incubation. *Key* List of labelled peaks is on Supplementary Table 8

In an attempt to analyze and identify any changes and modifications occurring to lysozyme in under the different formulation conditions, the supernatants were directly digested whilst the pellets (i.e. protein not in solution) were resolubilized by treating with 8 M urea before subsequent tryptic digestion and mass spectrometry analysis. These samples were compared to a tryptic digest of lysozyme in formulation 4 and to a standard protein digest. As shown in Fig. 4, there were observable changes between the peptide maps of the supernatants and the pellets after HPLC analysis separation alone. To identify those modifications that had occurred within the protein and where they had occurred, ESI–MS analysis of the tryptic peptides was undertaken (Fig. 5). Although mass data of good quality was collected, and each peptide peak could be assigned due to its mass, positive assignment of protein modifications proved difficult. While it was possible to identify the presence of different/extra peaks in the modified samples compared to the control samples it was not possible to match a mass to any known modification. The extra peaks present in digests consisted of all, or parts of, peptides corresponding to peptides T9, T15, T16, T12 + 13 and T11 (where T = peptide and the number = the tryptic peptide as expected from a theoretical tryptic digest from the *N*-terminal end of lysozyme (Povey et al. 2009)) and ms/ms analysis revealed that the masses of these extra peaks were not different from the expected masses of the peptides (Fig. 5 and Supplementary Table 8). It is possible that the modifications suppress the ionization of the modified form of the peptide, which means it is swamped by the unmodified material. Despite this, the data suggests it is within these regions of the protein covering the stretches within peptides T9, T15, T16, T12 + T13 and T11 that modification of the protein occurs.

### Mass spectrometry analysis of a model lysozyme peptide under variable formulation conditions

To be able to further investigate the amino acid residues that are prone to modification under formulation conditions, a peptide corresponding to residues T12 + T13 of chicken lysozyme was synthesized and incubated under the worst (formulations 1 and 12) and the best performing (formulation 4) formulations for the lysozyme samples. The reason for selecting the peptide from region T12 + 13 was that this was where most modifications have previously been observed to lysozyme under various conditions as reported in the literature (Smales et al. 2000; Povey et al. 2009). Further, the data presented above after tryptic peptide mapping and ESI–MS of the lysozyme samples in the different formulations also suggested this region of the protein is prone to modification. This peptide investigation allowed inspection as to whether such modifications are simply sequence dependent or whether they require a structural element.

After incubation or storage of the peptide in the different formulations for the appropriate time and temperature indicated, there was no detectable visual change in the appearance of the peptide in formulation 4. On-the-other-hand, after incubation of the model peptide in formulations 1 and 12 there was a large amount of yellow (formulation 1) and white (formulation 12) precipitate observed. After incubation of the peptide in formulation 1, no evidence of peptide dimerization as evidenced by the presence of a mass peak corresponding to a dimer was observed either in the supernatant or the pellet material (Supplementary Tables 9 and 10). The most abundant peak was at 1774 Da, a loss of 220 Da from the mass of the full peptide that equates to the loss of the first and the last amino acid residues from the peptide (serine and methionine) (Supplementary Tables 9 and 10). Loss of a water molecule was also observed in both the supernatant and the pellet material that is most likely due to a change to the proline residue (third residue) that is known to change conformation at high temperatures (Lu et al. 2008). Interestingly, after reduction with DTT both in the supernatant and the pellet a peak of 1791 Da was observed which equates to a loss of 203 Da from the intact peptide (Supplementary Table 9). This corresponds to loss of the first two amino acids in the sequence (serine and aspartic acid) suggesting that after incubation under these formulation conditions (formulation 1) and temperatures the peptide bonds can be broken and lead to peptide degradation.

The most abundant peak after incubation in formulation 12 for both the supernatant and the pellet material (3987 Da) was twice the expected mass of the peptide indicating that dimerization had occurred (Supplementary Table 9). After reduction the peak disappeared confirming that the dimerization was due to the cysteine residue forming disulphides. The second most abundant peak in both the supernatant and the pellet was the addition of two sodium ions (2037 Da, addition of 43 Da, Supplementary Table 9). After incubation in formulation 4, although no visual changes were apparent after incubation, the ESI–MS analysis showed the presence of a dimer peak (3985 Da) that after reduction with DTT disappeared indicating that this was from cysteine residue disulphide bond formation (Supplementary Tables 9 and 10).

## Conclusions

The buffer composition/concentration was the key variable of the formulation reagents in determining lysozyme stability and authenticity independent of protein concentration whilst the storage temperature and time, not surprisingly, were also key variables. Mass changes were observed that differed from the expected mass of lysozyme after incubation in some formulations that are therefore not suitable for storing lysozyme. Furthermore, the tryptic peptide mapping of the protein showed that the modifications occurred in the regions of the protein covering the stretches within peptides T9, T15, T16, T12 + T13 and T11 that have previously been reported to be prone to modification in the literature (Povey et al. 2009; Smales et al. 2000). The chicken lysozyme peptide from residues T12 + T13 was synthesized and incubated in such unsuitable formulations (formulations 1 and 12) and the best performing (formulation 4). The behavior of the peptide after incubation reflected the same behavior as the protein. Further the MS data showed that under the formulation conditions 1 (alkaline pH, high ionic strength) promoted peptide degradation while formulation conditions 12 (acidic pH, low ionic strength) favours dimerization. These findings suggest that peptide models could be utilized to mirror the stability of proteins or regions of proteins in the same formulations and could be used to help develop a rapid screen of formulations for stabilisation of troublesome elements and regions within biotherapeutic proteins.

## Electronic supplementary material

Supplementary material 1 (DOCX 37 kb)
