# Supplementary material for: Biotherapeutic protein formulation variables influence protein integrity and can promote post-translational modifications as shown using chicken egg white lysozyme as a model system
Source: Biotechnol Lett. 2015 Dec 23;38:589–96. doi: 10.1007/s10529-015-2014-y (PMC4796366; doi:10.1007/s10529-015-2014-y)
Supplement: Supplementary file 1 — Supplementary material 1 (DOCX 37 kb) [file 10529_2015_2014_MOESM1_ESM.docx]

**Supplementary Table 1. The variables investigated and the low/high amounts/value of each during Plackett-Burman Design analysis.**

| **Variables For lysozyme** | **Low level of variable** | **High level of variable** |
| --- | --- | --- |
| **Protein conc. (mM)** | 0.07 | 0.61 and 0.81 |
| **pH** | 8.5 * | 4 * |
| **Buffer conc. (mM)** | 500 | 10 |
| **Time (hrs)** | 168 | 2 |
| **Temperature (°C)** | 55 | 4 |
| **Glycine (M)** | 0.4 | 0.07 |
| **NaCl (M)** | 0.25 | 0.05 |

*For pH 8.5 sodium phosphate buffer, for pH 4.0 trisodium citrate buffer

**Supplementary Table 2. The Plackett-Burman seven variable factor design used to investigate the effects of formulation variables on protein integrity.**

| **Form.**  **No.** | **Protein Conc. (mM)** | **pH** | **Buffer Conc. (mM)** | **Time (hrs)** | **Temp (°C)** | **Glycine (mg/ml)** | **NaCl (M)** |
| --- | --- | --- | --- | --- | --- | --- | --- |
| **1** | High | High | High | High | High | High | High |
| **2** | Low | High | Low | High | High | High | Low |
| **3** | Low | Low | High | Low | High | High | High |
| **4** | High | Low | Low | High | Low | High | High |
| **5** | Low | High | Low | Low | High | Low | High |
| **6** | Low | Low | High | Low | Low | High | Low |
| **7** | Low | Low | Low | High | Low | Low | High |
| **8** | High | Low | Low | Low | High | Low | Low |
| **9** | High | High | Low | Low | Low | High | Low |
| **10** | High | High | High | Low | Low | Low | High |
| **11** | Low | High | High | High | Low | Low | Low |
| **12** | High | Low | High | High | High | Low | Low |

**Supplementary Table 3. Determination of lysozyme concentrations in solution after incubation in formulations 1-12 by measurement of A_280_ nm. The percentage of protein in solution was calculated by comparison to the pre-incubation concentration.**

| **Formulation** | **[lysozyme] before incubation (mg/ml)** | **[lysozyme] after incubation (mg/ml)** | **Standard deviation (n=3)** | **% lysozyme in solution after incubation** | **% lysozyme precipitated after incubation** |
| --- | --- | --- | --- | --- | --- |
| **Standard low (PBS, pH 7.2)** | 1.38 | 1.38 | 0.00 | 100 | 0 |
| **Standard high (PBS, pH 7.2)** | 11.39 | 11.39 | 0.01 | 100 | 0 |
| **1 (high)** | 8.63 | 4.99 | 0.70 | 58 | 42 |
| **2 (low)** | 1.09 | 0.88 | 0.04 | 81 | 19 |
| **3 (low)** | 1.01 | 0.92 | 0.10 | 91 | 9 |
| **4 (high)** | 9.48 | 9.34 | 0.15 | 98 | 2 |
| **5 (low)** | 1.55 | 1.53 | 0.00 | 99 | 1 |
| **6 (low)** | 1.45 | 1.33 | 0.01 | 92 | 8 |
| **7 (low)** | 1.48 | 1.25 | 0.02 | 84 | 16 |
| **8 (high)** | 9.53 | 9.47 | 0.04 | 99 | 1 |
| **9 (high)** | 10.67 | 10.55 | 0.15 | 99 | 1 |
| **10 (high)** | 8.77 | 8.35 | 0.12 | 95 | 5 |
| **11 (low)** | 1.20 | 1.15 | 0.04 | 96 | 4 |
| **12 (high)** | 6.94 | 6.55 | 0.13 | 94 | 6 |

**Key:** High/low in the formulation column refers to whether the formulation contained a low or high concentration of lysozyme protein.

**Supplementary Table 4. Statistical analysis of the effect of formulation variable on protein loss as determined by A_280_ analysis.** The statistical significance of changes in protein concentration (mg/ml) and % change levels relative to a PBS control for each variable are shown upon formulation and after incubation.

|  | **Significance of any change in soluble protein levels** | | | | |
| --- | --- | --- | --- | --- | --- |
| **Formulation variable** |  | **Upon formulation** | | **After Incubation** | |
|  |  | **mg/ml** | **%** | **mg/ml** | **%** |
| **Protein conc. (mg/ml)** | **p** | 0.008 | 0.262 | 0.289 | 0.986 |
|  | **d.o.f.** | 5 | 9 | 5 | 6 |
|  | **% sign.** | **99.2** | **73.8** | **71.1** | 1.4 |
| **Buffer comp. (mM)** | **p** | 0.304 | 0.155 | 0.309 | 0.433 |
|  | **d.o.f.** | 7 | 7 | 5 | 7 |
|  | **% sign.** | 69.6 | **84.5** | 69.1 | 56.7 |
| **pH** | **p** | 0.701 | 0.768 | 0.373 | 0.469 |
|  | **d.o.f.** | 9 | 7 | 5 | 6 |
|  | **% sign.** | 29.9 | 23.2 | 62.7 | 53.1 |
| **Temp. (°C)** | **p** | ­- | -­ | 0.390 | 0.342 |
|  | **d.o.f** | ­- | ­- | 5 | 6 |
|  | **% sign.** | ­- | ­- | 61 | 65.8 |
| **Time (hours)** | **p** | ­- | ­- | 0.319 | 0.157 |
|  | **d.o.f** | ­- | ­- | 5 | 5 |
|  | **% sign.** | ­- | -­ | 68.1 | **84.3** |
| **Glycine (mg/ml)** | **p** | 0.537 | 0.779 | 0.410 | 0.260 |
|  | **d.o.f.** | 8 | 9 | 5 | 6 |
|  | **% sign.** | 46.3 | 22.1 | 59 | 74 |
| **NaCl (M)** | **p** | 0.949 | 0.813 | 0.349 | 0.426 |
|  | **d.o.f.** | 9 | 8 | 5 | 6 |
|  | **% sign.** | 5.1 | 18.7 | 65.1 | 57.4 |

**Key:** d.o.f. = degrees of freedom, % sign. = level of statistical significance, p = p value from two-tailed student t-test.

**Supplementary Table 5. Lysozyme initial rates in formulations 1-12.** All values represent the average of three independent experiments (n=3). Standard formulations were prepared in PBS.

| **Formulation** | **Initial rate (Abs.units/sec)** | **Standard Deviation** |
| --- | --- | --- |
| **STD low**  **(1 mg/ml)** | 0.071 | 0 |
| **STD high (11.6 mg/ml)** | 0.048 | 0 |
| **1** | 0.016 | 0.002 |
| **2** | 0.051 | 0.004 |
| **3** | 0.043 | 0.005 |
| **4** | 0.046 | 0.003 |
| **5** | 0.053 | 0.008 |
| **6** | 0.049 | 0.004 |
| **7** | 0.057 | 0.005 |
| **8** | 0.048 | 0.002 |
| **9** | 0.047 | 0.000 |
| **10** | 0.044 | 0.009 |
| **11** | 0.038 | 0.005 |
| **12** | 0.025 | 0.006 |

**Supplementary Table 6. Lysozyme activity as determined by measurement of the initial rate of clearing of a bacterial solution in the various formulations and conditions investigated (n=3).** The percentage significance in terms of change relative to the control is shown.

| **Statistical testing of low versus high initial rates to determine changes to initial rate** | | |
| --- | --- | --- |
|  |  |  |
|  |  |  |
| **Formulation variable** |  | |
|  |  |  |
| **Protein conc. (mg/ml)** | **p** | 0.32 |
|  | **d.o.f** | 9 |
|  | **% sign.** | 68 |
| **Buffer conc. (mM)** | **p** | 0.084 |
|  | **d.o.f** | 6 |
|  | **% sign.** | **91.6** |
| **pH** | **p** | 0.606 |
|  | **d.o.f** | 7 |
|  | **% sign.** | 39.4 |
| **Temp. (°C)** | **p** | 0.746 |
|  | **d.o.f** | 8 |
|  | **% sign.** | 25.4 |
| **Time (hours)** | **p** | 0.612 |
|  | **d.o.f** | 5 |
|  | **% sign.** | 38.8 |
| **Glycine (mg/ml)** | **p** | 0.485 |
|  | **d.o.f** | 5 |
|  | **% sign.** | 51.5 |
| **NaCl (M)** | **p** | 0.236 |
|  | **d.o.f** | 7 |
|  | **% sign.** | 76.4 |

**Key:** d.o.f. = degrees of freedom, % sign. = level of statistical significance, p = p value from two-tailed student t-test.

**Supplementary Table 7. Enzymatic activity of lysozyme samples in different formulations determined using the bacterial substrate *Micrococcus lysodeikticus* (n=3)*.***

| **High conc. lysozyme formulation** | **STD high conc. in PBS** | **1** | **4** | **8** | **9** | **10** | **12** |
| --- | --- | --- | --- | --- | --- | --- | --- |
| **Initial clearing rate (Abs units/sec)** | 0.007 | 0.002 | 0.006 | 0.007 | 0.007 | 0.004 | 0.003 |
| **Maximum clearing rate (Abs units/sec)** | 0.025 | 0.003 | 0.010 | 0.008 | 0.011 | 0.007 | 0.004 |
| **[ *Micrococcus lysodeikticus* ] for 1/2 maximum clearing rate (mg/ml)** | 1.12 | 0.22 | 0.32 | 0.30 | 0.35 | 0.27 | 0.21 |

**Supplementary Table 8. Peptide peaks as labelled in Figure 5A with the observed mass of each peak and the corresponding assigned peptide (Povey et al 2009).**

| **Peak No.** | **Calculated mass (Da)** | **Observed mass (Da)** | **Peptide** |
| --- | --- | --- | --- |
| **1** | 606.4 | 606.5 | T1+T2 |
|  | 874.4 | 874.5 | T5 |
| **2** | 1428.65 | 1428.8 | T7 |
| **3** | 1050.5 | 1050.7 | T3+T4 |
|  | 894.4 | 894.5 | T3+T4 |
| **4** | 1492.6 | 1492.7 | T9+T10 |
|  | 994.4 | 994.6 | T9+T10 |
| **5a** | 1334.7 | 1334.7 | T15+T16 |
| **5b** | 1045.5 | 1045.7 | T16 |
| **5c** | 1753.83 | 1753.8 | T8 |
| **6** | 1326.6 | 1326.7 | T6 |
| **7e** | 1803.9 | 1803.8 | T12+T13 |
| **7f** | 1803.9 | 1804.6 | T12+T13 |
| **7g** | 1803.9 | 1804.6 | T12+T13 |
| **7h** | 1675.8 | 1676.8 | T13 |
| **7i** | 1675.8 | 1677.5 | T13 |
| **8j** | 2736.2 | 2736.6 | T6+T7 |
| **8k** | 2639.2 | 2639.4 | T11+T12 |
| **8l** | 2511.1 | 2511.4 | T11 |

**Supplementary Table 9. Mass analysis of the model lysozyme peptide and assignment of modifications after a week of incubation at 55°C in formulation 1 (supernatant and resolubilised (Res.) pellet), formulation 4 and formulation 12 (supernatant and resolubilised (Res.) pellet).**

| **Form. No** | **Mass observed (Da)** | **Peak Abundance (%)** | **Mass added/lost (Da)** | **Possible modification** |
| --- | --- | --- | --- | --- |
| **STD** | 1994 | 100 | - | peptide expected mass |
|  | 1975 | 22 | -18 | loss of water molecule |
| **1 supernatant** | 1976 | 22 | -18 | loss of water molecule |
|  | 1774 | 100 | -220 | loss of the first and last a.a. |
|  | 1976 | 59 | -18 | loss of water molecule |
|  | 1948 | 56 | -46 | ? |
|  | 2132 | 55 | +138 | ? |
|  | 1791 | 51 | -203 | ? |
| **1 Res. pellet** | 1774 | 100 | -220 | loss of the first and last a.a. |
|  | 1948 | 72 | -46 | ? |
|  | 1976 | 68 | -18 | loss of water molecule |
|  | 1791 | 58 | -203 | ? |
|  | 2150 | 33 | +156 | ? |
|  | 1433 | 33 | -561 | ? |
|  | 1993 | 33 |  | Peptide mass |
|  | 2132 | 29 | +138 | ? |
| **4 supernatant** | 1994 | 100 | - | peptide expected mass |
|  | 3985 | 94 | +1994 | dimer |
| **12 supernatant** | 3987 | 100 | +1994 | dimer |
|  | 2037 | 97 | +43 | disodium addition |
|  | 2053 | 56 | +58 | carboxymethylation on C***** |
|  | 2193 | 34 | +199 | ? |
|  | 2002 | 33 | +8 | ? |
|  | 1999 | 31 | +5 | ? |
|  | 1121 | 28 | -873 | ? |
| **12 Res. Pellet** | 3987 | 100 | +1994 | dimer |
|  | 2037 | 70 | +43 | disodium addition |
|  | 2193 | 46 | +199 | ? |
|  | 2053 | 27 | +58 | carboxymethylation on C***** |
|  | 1999 | 26 | +5 | ? |

**Key**: ? =unknown/unassignable, *****Cysteine reacts with iodoacetic acid to produce carboxymethyl cysteine.

**Supplementary Table 10. Mass analysis of the model lysozyme peptide after reduction of samples with DTT and assignment of modifications after a week of incubation at 55°C in formulation 1 (supernatant and resolubilised (Res.) pellet), formulation 4 and formulation 12 (supernatant and resolubilised (Res.) pellet).**

| **Form. No** | **Mass observed (Da)** | **Abundance peak (%)** | **Mass added/lost (Da)** | **Possible modification** |
| --- | --- | --- | --- | --- |
| **STD** | 1994 | 100 | - | peptide expected mass |
|  | 1976 | 22 | -18 | loss of water molecule |
| **1 reduced supernatant** | 1774 | 100 | -220 | loss of the first and last a.a. (S and M) |
|  | 1976 | 60 | -18 | loss of water molecule |
|  | 1791 | 51 | -203 | loss of the first two a.a. S and D (3-20) |
|  | 1993 | 30 | - | peptide expected mass |
|  | 1948 | 29 | -46 | ? |
|  | 1433 | 25 | -561 | ? |
| **1 reduced Res. pellet** | 1774 | 100 | -220 | loss of the first and last a.a. (S and M) |
|  | 1791 | 65 | -203 | loss of the first two a.a. S and D (3-20) |
|  | 1976 | 56 | -18 | loss of water molecule |
|  | 1948 | 47 | -46 | ? |
|  | 1433 | 35 | -561 | ? |
|  | 1993 | 30 | - | peptide expected mass |
|  | 1635 | 24 | -359 | ? |
| **4 reduced supernatant** | 1994 | 100 | - | peptide expected mass |
|  | 1977 | 11 | -18 | loss of water molecule |
| **12 reduced supernatant** | 1995 | 100 | - | peptide expected mass |
|  | 2011 | 26 | +16 | oxidation of M |
| **12 reduced Res. Pellet** | 1995 | 100 | - | peptide expected mass |
|  | 2011 | 24 | +16 | oxidation of M |

**Key**: ? =unknown/unassignable
